# The prevalence and diagnostic performance of anti-cyclic citrullinated peptide antibody in rheumatoid arthritis: the predictive and discriminative ability of serum antibody level in recognizing rheumatoid arthritis

**DOI:** 10.4103/0256-4947.57170

**Published:** 2009

**Authors:** Behzad Heidari, Alireza Firouzjahi, Parnaz Heidari, Karim Hajian

**Affiliations:** a From the Department of Medicine, Babol University of Medical Sciences, Babol, Mazandran, Iran; b From the Department of Pathology, Babol University of Medical Sciences, Babol, Mazandran, Iran; c From the Department of Social Medicine, Babol University of Medical Sciences, Babol, Mazandran, Iran; d From the Department of Faculty of Medicine, Azad University of Tehran, Tehran, Iran

## Abstract

**BACKGROUND AND OBJECTIVES:**

The utility of anticyclic citrullinated peptide (anti-CCP) antibody in the diagnosis of rheumatoid arthritis (RA) varies across different studies. We determined the diagnostic performance and predictive ability of anti-CCP for RA.

**METHODS:**

We studied 201 patients with RA and compared them with 208 non-RA patients as controls. RA patients included in the study fulfilled the American College of Rheumatology revised criteria and patients with other diseases as well as those with undifferentiated arthritis (UIA) were used as controls. Anti-CCP was measured by enzyme-linked immunosorbent assay (ELISA) and rheumatoid factor (RF) by the agglutination method. The optimal cutoff value and diagnostic accuracy were determined using receiver operating characteristics (ROC) curve and area under the curve (AUC).The sensitivity and specificity were determined by comparison of RA patients with non-RA controls.

**RESULTS:**

The anti-CCP test was positive in 164 patients with RA for a sensitivity of 81.6%, specificity of 87.5%, and overall accuracy of 84.6%. The respective values for RF were 75.6%, 86.5% and 84.4%. The anti-CCP test discriminated RA from non-RA patients with high accuracy (AUC=0.889 [0.017] 95% CI, 0.856-0.952, *P*=.001), and predicted progression of UIA to RA with moderate accuracy (AUC=0.733 [0.069], 95% CI 0.60-0.87, *P*<.006) at a sensitivity of 75% and a specificity of 68.1%. Among 60 UIA patients, in 16 (26.7%) who differentiated to RA, the mean (standard deviation) for anti-CCP was significantly higher than in 24 (40%) patients who progressed to non-RA (134.8 [172] vs 46 [86] U/mL, *P*<.01).

**CONCLUSION:**

These findings indicate that anti-CCP yields higher sensitivity and overall accuracy, but slightly greater specificity than RF for diagnosis of RA. Anti-CCP positivity, particularly a higher level of serum antibody in patients with UIA, may be a predictor of subsequent RA.

Rheumatoid arthritis (RA) is a chronic inflammatory autoimmune disease with a progressive course that may lead to joint destruction and subsequent disability if not effectively treated.^1^ Early diagnosis and treatment may prevent joint destruction and suppress disease progression.^2^

At present, the diagnosis of RA is based on the American College of Rheumatology (ACR) revised criteria, which include IgM rheumatoid factor (RF), and clinical and radiological criteria.^3^ However, RF positivity is nonspecific for RA, because it can be detected in other non-RA diseases as well as in healthy individuals. The initial diagnosis of RA at the initial period can be difficult in some cases due to lack of typical symptoms and signs to fulfill the ACR criteria. Therefore, the presence of a sensitive and specific test for diagnosis of RA at an early stage would be very useful for initiating treatment as well as in discriminating RA from non-RA diseases. Among the several autoantibodies described for diagnosis of RA, anticyclic citrullinated peptide (anti-CCP) antibodies yielded a higher specificity with comparable or even higher sensitivity compared with RF.^4^–^8^

Anti-CCP antibodies are produced locally at sites of inflammation, not only in the synovium of RA, but also in other non-RA diseases.^9^ Therefore, anti-CCP positivity may be expected in a proportion of patients with non-RA diseases as well. Hence, discrepancy in sensitivity and specificity of anti-CCP between various studies may be attributed to differences in false-positive rates among selected controls. However, other factors such as detection techniques and genetic background may be also responsible for these variations. For this reason, the present study was designed to determine the diagnostic performance of anti-CCP in patients who presented to an outpatient medical clinic in Babol, Iran.

## METHODS

All patients were selected consecutively over a 1-year period from among patients visiting rheumatology clinics in Babol, Iran. Patients with inflammatory arthritides who had one or more inflamed joints were included in this study. The study was approved by the ethical committee of Babol University of Medical Sciences. RA patients included in the study fulfilled the ACR revised criteria; non-RA patients considered as controls fulfilling other criteria for other non-RA rheumatic diseases.

A medical history and complete clinical, laboratory and radiological examination were performed on all patients. Initially, 185 patients diagnosed as having RA and 50 were non-RA. Sixty patients with a mean (SD) disease duration of 2.3 (3.1) years, who did not fulfill any diagnostic criteria, were classified as having undifferentiated inflammatory arthritis (UIA).

All patients were treated appropriately and followed for at least 1 year or longer. Patients visited every 2 to 3 months. The initial diagnoses were reviewed or changed when there were new clinical, laboratory, or radiological findings. Final diagnoses of RA or non-RA, which were made at the latest visit, were considered in the statistical analysis.

Serum samples were obtained for assessment of anti-CCP and RF. Anti-CCP levels were determined by ELISA using the Euroimmune kit, which measures human IgG antibody against anti-CCP. RF was assessed by the latex agglutination method. Additionally, sera from 114 apparently healthy subjects without arthritis were tested to assess the specificity of anti-CCP and RF.

Receiver operating characteristics (ROC) analysis was applied by plotting sensitivity against 1-specificity for various cutoff points of anti-CCP. The optimal cutoff value that best distinguished RA from non-RA was determined at the maximum value of Youden's index, which was estimated by sensitivity + 1-specificity.^10^ The overall diagnostic accuracy and predictive ability were estimated based on the area under the curve (AUC) which is reported with its standard error.

Considering clinical and radiographic criteria as the gold standard diagnostic test, the diagnostic characteristics were determined by comparison of RA with non-RA controls. Controls consisted of patients with non-RA inflammatory arthritides and 114 subjects who attended the same clinic and were selected consecutively among those without any skeletal diseases. The misclassification rate (MR) was calculated by false negative + false positive divided by the total of the tests performed. Accuracy was calculated by 1–MR and likelihood ratio was calculated by sensitivity divided by 1–specificity.

## RESULTS

Two hundred ninety-five patients entered the study (Table 1). After a median follow up of 14 months (range 1-50), 16 of 60 UIA patients met the ACR criteria for RA, 8 patients progressed to SLE, and 18 patients progressed to rheumatic diseases other than RA. In the remaining 18 patients, the diagnosis did not change. The study included 201 RA patients (80% females) with mean age of 51 (14) years with a mean disease duration of 6.6 (6.3) years, 94 non-RA patients with a mean disease duration of 2.6 (4) years and 114 subjects without arthritis with mean age of 39 (16) years who served as controls.

**Table 1:** Frequency of patients with rheumatoid arthritis and other rheumatic diseases at presentation and over the follow-up period.

Diagnosis	No. of patients	
	Initial diagnosis	Final diagnosis
RA	185	201
UIA	60	18
Non-RA inflammatory arthritis	50	76
Healthy controls	114	114

**Total**	**409**	**409**

**Figure 1:**
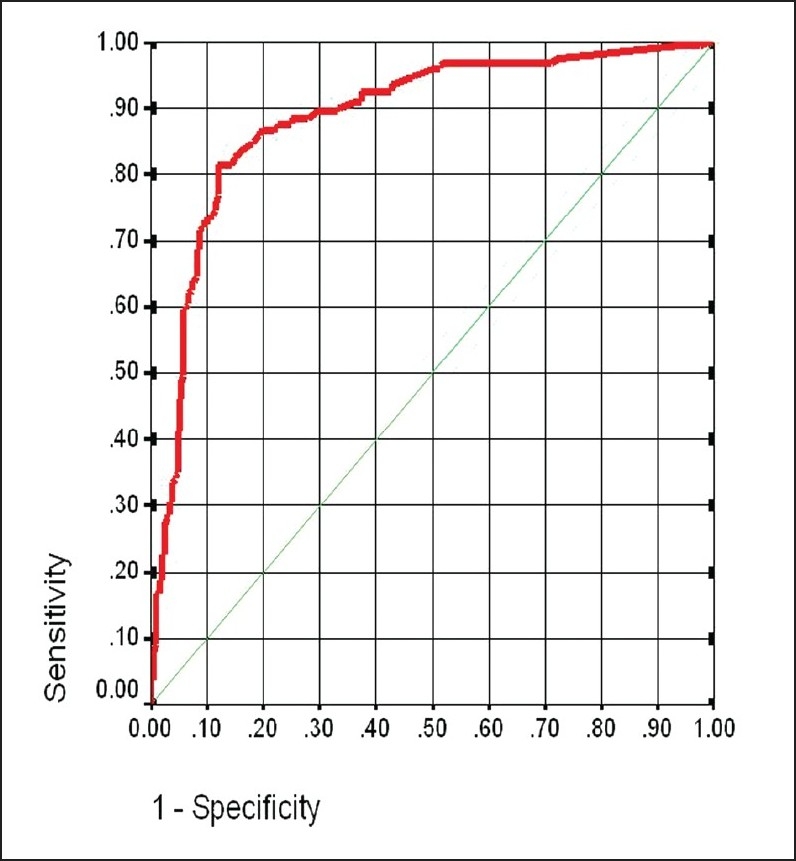
Receiver operating characteristics curve for anti-CCP antibody in the diagnosis of RA.

Based on the results of ROC analysis, a serum anti-CCP of 14.8 U/mL yielded the highest Youden's index value for diagnosis of RA at a sensitivity of 81.6%, a specificity of 87.5%, and overall accuracy rate of 84.6% (Figure 1). At this level the anti-CCP test demonstrated a false positive rate of 12.5%, a false negative rate of 18.4%, a misclassification rate of 15.4% and a positive likelihood ratio of 6.52. Anti-CCP diagnosed RA and differentiated RA from non-RA with high diagnostic accuracy with an AUC value of 0.889 (0.017) none (95% CI, 0.856-0.952, *P*=.001). Patients with initial UIA who subsequently progressed to RA or non-RA were differentiated by anti-CCP at a sensitivity of 75% and a specificity of 68.1% with a moderate accuracy at AUC of 0.733 (0.069) (95% CI 0.60-0.87, *P*<.006). Anti-CCP was positive in 32 (65.3%) seronegative RA patients. Diagnostic characteristics of anti-CCP and RF are presented in Table 2.

**Table 2:** Diagnostic characteristics of anti-CCP and rheumatoid factor for diagnosis of rheumatoid arthritis.

Diagnostic tests	Sensitivity	Specificity	Misclassification	Accuracy	Likelihood ratio
Anti-CCP	81.6	87.5	15.4	84.6	6.52
RF	75.6	86.5	18.8	81.2	5.6
Anti-CCP and RF	65.6	95.2	19.3	80.5	13.6

anti-CCP=anticyclic citrullinated peptide antibody; RF=rheumatoid factor

The mean (SD) antibody levels in anti-CCP-positive RA, SLE, and palindromic arthritis, patients were 139 (118), 121 (157), and 130 (94) U/mL, respectively, but the mean anti-CCP in 16 patients with UIA who progressed to RA was significantly higher than in the 24 UIA patients who progressed to non-RA (135 (172) U/mL vs. 46 (86) U/mL, *P*=.011). RA development in anti-CCP-positive UIA was 6.5 times higher than anti-CCP-negative UIA (46.1% vs. 11.7%, *P*<.002).

## DISCUSSION

We estimated the diagnostic performance and overall accuracy of anti-CCP, and explored the relationship between serum antibody level and progression of arthritis to RA. The results of this study suggest that anti-CCP is a better diagnostic tool than RF for diagnosis of RA with higher sensitivity, only a slightly greater specificity, but a higher diagnostic accuracy. The presence of both antibodies in the serum compared with anti-CCP alone increases the specificity, but decreases both sensitivity and overall accuracy. Moreover, anti-CCP displays a high diagnostic accuracy in discriminating RA from non-RA patients and a moderate accuracy in predicting progression of UIA to RA.

Available data indicate variations in sensitivity and specificity of anti-CCP across different studies.^4^–^8^ Based on a meta-analysis of 37 studies of anti-CCP antibody and 50 studies of RF by Nishimura et al, anti-CCP was more specific than RF for diagnosing RA. The pooled sensitivity, specificity, and positive likelihood ratio for anti-CCP antibody were 67%, 95%, and 12.46, and for IgM RF the values were 69%, 85%, and 4.86, respectively.^11^ Among several factors that could explain the discordant sensitivity and specificity between diverse studies, the frequency of a false positive test in non-RA controls deserves further consideration because the specificity is inversely related to the proportion of positive tests among controls. The lower specificity of anti-CCP in the present study compared with other studies can be explained by the presence of a high proportion of false positive non-RA arthritis among controls. In contrast, in studies in which healthy subjects or noninflammatory arthritis patients were selected as controls, the specificity was higher because the likelihood of anti-CCP positivity in healthy subjects or noninflammatory arthritis is lower than in patients with inflammatory arthritides.^6^–^8^

In another study from Iran of 136 RA patients with a disease duration of less than two years, the sensitivity and specificity of anti-CCP were 62.5% and 89.1%, respectively.^5^

In the present study, not only the presence of anti-CCP in the serum, but the level of antibody in the serum, was also associated with subsequent progression of UIA to RA. As yet, the relationship between the level of serum antibody and progression to RA was shown only in one previous study.^12^ The results of our study confirm previous results, provide further data, and extend evidence-based knowledge. Based on the results of the present study, patients with anti-CCP-positive inflammatory arthritis and patients with a high level of serum antibody, in particular, may progress to RA afterwards.

A important point that could be seen as affecting the interpretation of this study is that palindromic arthritis is considered a risk factor for RA and anti-CCP-positive SLE may develop clinical features similar to RA. Therefore, in a number of anti-CCP-positive non-RA patients, differentiation to RA with subsequent changes in sensitivity and specificity is possible.^13^,^14^ However, the overall results would not be affected significantly.

In conlusion, in Iranian patients, anti-CCP compared to RF yields a higher sensitivity with slightly greater specificity, but higher overall accuracy for diagnosis of RA. Anti-CCP differentiates RA from non-RA with high accuracy. Anti-CCP positivity, particularly a higher level of serum antibody, may predict progression of UIA to RA. Further studies are required to confirm the accuracy as well as the predictive ability of serum antibody level for subsequent RA.
